# The *why* of the phenomenal aspect of consciousness: Its main functions and the mechanisms underpinning it

**DOI:** 10.3389/fpsyg.2022.913309

**Published:** 2022-07-28

**Authors:** Giorgio Marchetti

**Affiliations:** Mind, Consciousness and Language Research Center, Alano di Piave, Italy

**Keywords:** information, phenomenal aspect of consciousness (PAC), attention, energy, the self (S), organ of attention (OA)

## Abstract

What distinguishes conscious information processing from other kinds of information processing is its phenomenal aspect (PAC), the-what-it-is-like for an agent to experience something. The PAC supplies the agent with a sense of self, and informs the agent on how its self is affected by the agent’s own operations. The PAC originates from the activity that attention performs to detect the state of what I define “the self” (S). S is centered and develops on a hierarchy of innate and acquired values, and is primarily expressed via the central and peripheral nervous systems; it maps the agent’s body and cognitive capacities, and its interactions with the environment. The detection of the state of S by attention modulates the energy level of the organ of attention (OA), i.e., the neural substrate that underpins attention. This modulation generates the PAC. The PAC can be qualified according to five dimensions: qualitative, quantitative, hedonic, temporal and spatial. Each dimension can be traced back to a specific feature of the modulation of the energy level of the OA.

## Introduction

Various different theories try to explain the underlying mechanisms of consciousness (for recent reviews, see Northoff and Lamme, 2020; Winters, 2021). One of the most promising approaches that is fully adopted or partly shared by some of these theories is to investigate consciousness in informational terms. Chalmers (1996, pp. 285–287) explicitly theorized that information can be a good construct to make the link between physical processes and conscious experience. Since then, the idea that consciousness can be investigated in informational terms, has been recurrently put forward both in scientific research and philosophical debates (Tononi, 2008, 2012; Aleksander and Gamez, 2011; Earl, 2014; Jonkisz, 2015, 2016; Tononi and Koch, 2015; Fingelkurts and Fingelkurts, 2017; Orpwood, 2017; Ruffini, 2017; Marchetti, 2018; Kanai et al., 2019; but some researcher had adopted this idea even before Chalmers’ proposal: see Baars, 1988).

While generally supported by theoretical considerations concerning the nature of life^1^, this approach is not exempt from criticism, above all for its panpsychist implications (see for example Pockett, 2014).

I endorse such an approach as my starting point, but I maintain that not all kinds of information processing are conscious: after all, there is ample evidence of information processed by humans unconsciously, as well as of not-conscious information processed by computers. Consciousness is a phenomenon that evolved by purely biological processes on a planet where it did not exist previously (and that, once appeared, can theoretically also be artificially replicated).

Given this, the fundamental question to be addressed is what distinguishes conscious information processing from other kinds of information processing.

The answer is to be found in what primarily distinguishes consciousness from other phenomena: the qualitative, *phenomenal aspect of consciousness* (from here on: PAC), i.e., the what-it-is-like for an agent to experience something. Taking the PAC into consideration, what it adds to information processing^2^, and, above all, what difference it makes to the agent that is processing information, allows one to understand what distinguishes conscious information processing from other kinds of information processing. Ultimately, this means explaining the *why* of the PAC: why it is needed for an agent and why it has the form it has.

It can certainly be argued that the difference between conscious information processing and other kinds of information processing can also be found somewhere else than in the PAC, for example, in the specific organization and functioning of the brain as compared to the organization and functioning of systems performing a different kind of information processing (Fingelkurts et al., 2010, 2013; Rabinovich et al., 2012; Fingelkurts and Fingelkurts, 2017).

According to Fingelkurts and Fingelkurts (2017, p. 2), the brain is “an active system that retains the characteristics of a complex, non-linear system with non-equilibrium dynamics, reflected in transient evolution of transient states in the form of discrete frames of activity and phase transitions between micro- and macro-levels.” As such, the information processed by the brain is characterized by self-organization, the interplay of stability/instability, timing of sequential processing, coordination of the multiple sequential streams, circular causality between bottom–up and top–down operations, and information creation, all aspects that cannot be captured by the classical Shannonian concept of information. Consequently, the information processed by the brain (as opposed to the information processed by other systems) can be described as “ordered sequences of metastable states across multiple spatial and temporal scales.” In a similar vein, Rabinovich et al. (2012, pp. 51, 60) maintain that “brain flow information dynamics deals with problems such as the stability/instability of information flows, their quality, the timing of sequential processing, the top–down cognitive control of perceptual information, and information creation” and consequently that a cognitive (mental) information flow can be defined as “a flow along a chain of metastable states.”

I think that distinguishing conscious information processing from other kinds of information processing on the basis of the organization and functioning of the brain certainly offers an important contribution to the explanation of salient aspects of consciousness, such as the stream of consciousness and how the brain creates new information. However, without a preliminary analysis of the PAC, of the difference it makes for information processing and for the agent processing it, one can hardly capture the core difference between conscious information processing and other kinds of information processing: after all, what primarily distinguishes consciousness from all other phenomena is its qualitative, phenomenal aspect. Moreover, one must also take the PAC into consideration whenever one wants to deal with the other features of consciousness (such as the stream of consciousness or the unity of a conscious state): in fact, it is principally on the basis of the PAC that one can identify these features.

Following Chalmers (1995), it can also be argued that the way in which I tackle the problem of the PAC (that is, by relating the PAC to the agent: “What difference does it make to the agent that is processing information?”) will never help solve the hard problem of consciousness (“Why and how do physical processes in the brain give rise to experience?”) because phenomenal consciousness and states are not relational phenomena (they cannot be functionally defined) but intrinsic ones (Di Francesco, 2000). I think that the distinction between the hard problem of consciousness and the easy problem of consciousness is misleading because it creates a break where there is none. Phenomenal consciousness and conscious phenomena are such simply because there is an experiencing subject (the agent) who experiences them, and to whom they make a certain difference (that is, they have a certain meaning for the experiencing subject). An experience without its experiencing subject is no longer an experience: it loses its meaning. As Nida-Rümelin (2017, p. 56) observes: ‘‘we cannot even think the occurrence of an experience without thereby thinking of it as involving an experiencing subject.’’ The experience of pain is such because there is an experiencing subject who experiences it: without the experiencing subject, there will be only an empty, abstract concept - the concept of ‘‘pain’’ - but no actual experience of pain. When I say that I have toothache, you can certainly understand what I mean, but you cannot feel my toothache (or, said otherwise, you experience the meaning of the word ‘‘toothache’’ but you do not experience any toothache). In sum, the problem of the PAC can only be solved by also taking its experiencing subject into account^3^.

Therefore, in this article I aim principally at answering the question of the *why* of the PAC, that is, the difference that it makes for the agent that processes information. I will then offer a tentative explanation of the mechanism that underpins the PAC. For reasons of space, I will not deal with higher forms of consciousness, such as meta-cognitive consciousness. Suffice it to say that most arguably these higher forms of consciousness are made possible by, and develop on, the more basic form of phenomenal consciousness, once reflective self-awareness has formed (Gallagher and Zahavi, 2008).

## Current research on consciousness deals principally with the *how*, not with the *why* of the phenomenal aspect of consciousness

Generally speaking, two main approaches are adopted when analyzing consciousness in informational terms. The most common one is to take the PAC for granted, without directly investigating its *why*, that is, its role in an agent’s processing of information. For example, Ruffini (2017, p. 2) clearly states: “We do not address here the *hard problem* of consciousness – the fundamental origin of experience (…) We assume that *there is consciousness*, which, with the right conditions, gives rise to structured experience”. Likewise, Kanai et al. (2019, p. 2) maintain that “Instead of directly addressing the Hard problem, a possibly more productive direction might be to consider putative functions of consciousness, namely, cognitive functions that require consciousness in the sense of being awake and able to report stimulus contents with confidence.”

This approach primarily investigates the *how* of consciousness, that is, what structures bring it about, such as the neural correlates of consciousness (NCC) (e.g., Baars, 1988; Mashour et al., 2020) and the brain’s internal models of the environment that allows agents to simulate the consequences of their own or other agents’ actions and avoid dangerous outcomes (e.g., Ruffini, 2017; Kanai et al., 2019). This approach also focuses on the possible functions that consciousness may have in supporting the other cognitive functions (e.g., the executive one). However, strangely indeed, the functions of consciousness are mostly considered and explained independently of the PAC. Even when somehow relating the function of consciousness to the PAC, scholars adopting this approach do not consider the role that the PAC plays in processing information. For example, various scholars claim that consciousness has the function of making information globally available across the system, and transforming data into a *format* that can be easily and flexibly used by high-level processors (language, autobiographical memory, decision-making, metacognition, etc.) (Baars, 1988; Dehaene and Naccache, 2001; Earl, 2014; Mashour et al., 2020; Frigato, 2021). However, they do not explain why this must be the case, that is, why only information and data that have the particular phenomenal aspect that consciousness assigns to them, can be made globally available across the system and processed by high-level processors. Kanai et al. (2019, p. 6), in their “information generation” model of consciousness, recognize the importance of the PAC when they observe that it allows for distinguishing representations of factual reality of the here and now from counterfactual representations (e.g., past and future events), because the former are more vivid than the latter. They also explain that the difference in vividness of experience comes from the difference in the degree of details produced by the generative model that they have postulated. However, they completely skip the essential question: Why is the PAC needed to distinguish representations of factual reality from counterfactual representations? Could an agent not make such distinctions unconsciously? What does experience (of vividness as well as of anything else) do that the lack of experience cannot do?

The second kind of approach does try to account for the PAC, but, similarly to the first, it focuses mostly on the *how* of conscious experience instead of the *why*. Therefore, this approach is not of great help either in explaining the role that the PAC plays in an agent’s processing of information, as well as in differentiating conscious information processing from other kinds of information processing. As an example of this kind of approach, let’s briefly consider the Integrated Information Theory of consciousness (IIT) put forward by Tononi (2008, 2012), Oizumi et al. (2014), and Tononi and Koch (2015). IIT directly tackles the PAC: it firstly identifies the main phenomenological properties of consciousness, what IIT defines as “axioms”: intrinsic existence, composition, information, integration and exclusion. Then it derives a set of “postulates” that parallel the axioms and specify how physical systems might realize these axioms. Last, it develops a detailed mathematical framework in which the phenomenological properties are defined precisely and made operational. IIT defines consciousness as integrated information (Φ), where integrated information stands for the amount of information generated by a complex of elements, above and beyond the information generated by its parts.

The choice of IIT to limit the investigation of consciousness to its phenomenological properties limits IIT’s possibilities to explain the role that the PAC plays in an agent’s processing of information. IIT considers the phenomenological properties of consciousness in themselves, without any connection to the possible cognitive functions they can have (such as planning and initiation of behavior). This choice, which has led Cerullo (2015) to define IIT as a theory of “protoconsciousness” or “non-cognitive consciousness” (as opposed to a theory of “cognitive-consciousness”), makes IIT tackle a kind of consciousness that substantially differs from the one tackled by psychology, cognitive neuroscience and neurology. While the latter is supposed to have evolved in association with the other cognitive functions of the system (such as memory and attention) in order to assist the system in controlling its own behavior, the former does not necessarily imply a functional role for the system’s behavior, and lacks the cognitive properties associated with such a role. Indeed, IIT does not intend to explain why, to what purpose a system should generate phenomenal consciousness. Rather, IIT intends to explain how the generation of integrated information leads to the PAC. In sum, this limits IIT’s possibilities to account for the possible functions of the PAC, as well as for the functions of the other cognitive functions of the system (memory, attention, etc.) associated with the PAC.

A related argument has been put forward by Safron (2020), according to whom most of IIT’s problems originate from the fact that IIT does not take the agent’s interactions with the world into consideration (“Without those meaningful external connections, systems could have arbitrarily large amounts of integrative potential, but there still may be nothing that it is like to be such system,” Safron, 2020, p. 16). In Safron’s view, the minimal condition for a system to be conscious is that it is capable of generating, from an egocentric perspective, integrated system-world models with spatial, temporal, and causal coherence, all of which require agentic, autonomous selfhood. Consequently, he suggests integrating IIT (and GNWT) with the Free Energy Principle and Active Inference Framework (FEP-AI) (Friston et al., 2006, 2017), which provides a formalism of how internal states can model external states. While I agree with Safron in that, in order to deal with consciousness, it is necessary to take the system’s interactions with the world into account (see my discussion on the sense of self in the section “Why is the phenomenal aspect of consciousness needed?”), I think that FEP-AI, albeit being useful in defining how a system’s internal states can model external states, is of limited utility in explaining the basis on which the distinction between the system (or self) and the world takes place. As Di Paolo et al. (2022, p. 28) explain, FEP-AI presupposes such a distinction, instead of explaining it: “All processes subserving self-distinction are themselves products of self-production. In contrast, Markov blankets in FEP systems are there by assumption (…) there is nothing in the Markov Blanket that necessarily links it to processes of organismic constitution.” As it will become clear later in the article, in order to account for the basis of the system-world distinction, it is necessary to consider the attentional mechanism underlying the hedonic dimension of the PAC.

The integrated information theory of consciousness has also raised some other criticisms because of its identification of consciousness with integrated information. Taken to extremes, this identification leads one to maintain that any system that has integrated states of information is conscious (Cerullo, 2015; Jonkisz, 2015), which implies some counterintuitive consequences, such as the attribution of consciousness to simple artifacts, such as photodiodes.

Moreover, as Mudrik et al. (2014) show, there are at least four integrative processes that occur without consciousness, that is, short-range spatiotemporal integration, low-level semantic integration, single sensory (versus multisensory) integration, and previously learned (versus new) integration. Therefore, information integration, even if it turns out that it is most probably necessary for consciousness, is not sufficient.

Finally, Mindt (2017) observes that, even though IIT can provide a detailed account of how experience might arise from integrated information, it nevertheless leaves open the question of why it feels like something for a brain to integrate information.

Similar to IIT, the theory put forward by Orpwood (2017) also tackles directly the PAC. Orpwood argues that qualia are a likely outcome of the processing of information in local cortical networks: qualia would arise when attention or some other re-entrant processes develop an attractor state in a network that enables the network to identify the information cycled (at least, three times) through it as a representation of the identity of its previous input. However, Orpwood does not explain why, to what purpose, consciousness is required to perform such an identification process: could such an identification not occur without the support of consciousness?

It is anyhow important to note that, despite their inability to explain the why of the PAC, the majority of theories developed within these two approaches do provide powerful tools in the scientific study of consciousness, both in terms of their predictive capacity, testability and possibility of carrying out precise measures. For example, GNW (Global Neuronal Workspace) (Mashour et al., 2020, p. 789) very clearly predicts that consciousness can be disrupted when the function of cortical hubs or reverberant connectivity is disrupted.

## Why is the phenomenal aspect of consciousness needed?

Let’s now try to answer the fundamental questions about the PAC.

To begin with: what difference does the PAC make in general? No doubt, the PAC makes experience appear as it is – that is, an “experience” – and makes it differ from other experiences: it makes factual reality appear what it is – that is, “real” – and makes it differ from dream; it makes pain appear as “painful” or “hurting” and pleasure as “pleasant”; it makes pain differ from pleasure, and a big pain differ from a small pain.

But why is the PAC needed at all? As the philosopher Campbell (2011, p. 323) asked: “Why should *experience* be needed? Why not just any way of being causally impacted by the events around us, in a way that gives information about them?” Could we not do without experience and process the information unconsciously? After all, much – if not the majority - of what happens inside our brain, happens without our knowledge.

My answer is that *the information provided by the PAC supplies the agent with a sense of self, and how this self is affected by the agent’s own operations^4^*.

Let’s clarify how the terms “operation,” “information,” “sense of self,” and “affected” must be understood.

By “operations” (and “operate”) I refer in a very general sense to the various physical and mental activities that an agent performs, either on an active, voluntary, goal-directed basis (e.g., walk, eat, speak) or on a passive, involuntary, stimulus-driven one (e.g., dream, involuntarily move in response to a stimulus, perceive pain, feel hungry or thirsty).

By “information” (and “inform”) I do not refer to a “universal” kind of computation, according to which information can be programmed in, and represented by any abstract symbols, but rather to a “fixed” kind of computation, according to which the hardware and software are interdependent, and information is instantiated in the form of the structure (Farnsworth et al., 2013). While in the former kind of computation, information can be instantiated by any program, code and physical system, in the latter (which is typical of living systems), information can only be instantiated by the specific biological system (or agent) that embodies it. This has three important implications. The first is that the agent does not need at all to decode, translate or transduce the message of the PAC into any other language: the agent immediately grasps the message of the PAC by experiencing it, because what the PAC means, its content, coincides with the form (the aspect) of the PAC (Marchetti, 2018). This happens even when the message is ambiguous, lacks sufficient clarity or just provides a very general feeling, such as that of rightness or familiarity: in fact, in such cases, the meaning conveyed by the conscious experience is precisely of ‘‘ambiguity,’’ ‘‘not sufficiently clear,’’ ‘‘familiarity,’’ etc.^5^ The second implication is that the precise meaning that the information provided by the PAC has for the agent that experiences it, can only be understood by the agent itself and not by another agent: in other words, I know what it means for me to experience “pain,” but another person cannot directly know what it means for me to experience “pain” (and vice versa). This is because the information provided by conscious experience is always “individuated” – to use Jonkisz’s (2015) expression –, that is, it is shaped by the agent’s evolutionary antecedents and by its unique and particular interactions with its environment and other agents. The third implication is that the information provided by the PAC cannot be adequately dealt with by every theory of information. This is because the PAC is derivative on an experiencing subject who produces and interprets it. Therefore, those theories of information - such as Dretske (1981); Floridi (2005), and Mingers and Standing’s (2014) – which maintain that information is fundamentally objective and exists independently of the agent that produces it, cannot adequately account for the information provided by the PAC. A more suitable theory of information seems to be Hofkirchner’s (2013, 2014) unified theory of information (UTI), because it shows how (self-organizing) systems produce information. According to Hofkirchner (2013, p. 9), information is produced when “self-organizing systems relate to some external perturbation through the spontaneous build-up of order they execute when exposed to this perturbation.” Self-organizing systems produce information because they transform the input into an output in a non-deterministic and non-mechanical way. On the contrary, computers, probabilistic machines and other systems that compute and work according to strict deterministic rules, which by definition do not yield novelties, cannot produce information (Hofkirchner, 2011). Hofkirchner’s definition of information production can be equated with Bateson’s (1972) famous definition of information as a “difference which makes a difference.” Bateson’s “making a difference” is the build-up of the system’s self-organized order; Bateson’s “difference” that makes a difference is a perturbation in the inner or outer environment of the system that triggers the build-up; Bateson’s “difference that is made” is made to the system because the perturbation serves a function for the system’s self-organization.

The “sense of self” can be described as characterized by the following fundamental features: (a) the sense of being an entity differentiated from other entities. This provides the agent with a sense of mineness or ownership, that is, the quality that all its experiences belong to, and are for it (and not for-someone-else); (b) what can be defined as the “point of view” from which any content is “seen”. This point of view persists through all conscious experiences independently of their contents (Winters, 2021, p. 12) and partitions the world into the asymmetric space of what monitors and what is monitored (Merker, 2013a,b); (c) a feature that is strictly associated with the “point of view”: the feeling of continuity. Our experience flows uninterruptedly like a river. As James (1890/1983, pp. 233–234) observed: “the transition between the thought of one object and the thought of another is no more a break in the thought than a joint in a bamboo is a break in the wood.” The feeling of continuity is assured even when there are temporary interruptions in conscious experience (because of sleep, anesthesia, etc.): indeed, these interruptions are not experienced directly as such, that is, as gaps of consciousness, but indirectly, as conscious experiences of having lost consciousness. As Evans (1970, p. 185) observed : “It is only by inference that we know that we have been unconscious, or by being told of this by someone else.” That is, the sense of self acts like an uninterrupted, permanent background on which specific, separated contents follow one another, and changes can be perceived; (d) last but not least, the capacity it has to represent an organism composed of multiple, interconnected parts in the unified and condensed way of a “single voice” (Damasio, 2010), that is as a single unit. This allows the agent to devise plans and actions that best fit its existence as a whole, rather than favoring some of its parts to the detriment of the other ones, and coordinate its behavior accordingly: in a word, to maintain and expand the well-being of the agent in its entirety.

It could be claimed that exceptional conscious states – such as those induced by drugs or meditation, and pathological conscious states – may lack some of the features that a sense of self implies (e.g., spatial self-location, mineness), if not all of them. After all, these states often present a phenomenology that substantially differs from the phenomenology of ordinary conscious experience. Consider, for example, the alleged cases of self-loss or ego-dissolution reported by highly experienced mindfulness meditators: “it’s like falling into empty space…and a sense of dissolving […] there’s no personal point of view, it’s the world point of view, it’s like the world looking, not [me] looking, the world is looking” (Millière et al., 2018, p. 11), or by users of psychedelic drugs: “I wasn’t anything anymore. I had been broken down into nothingness, into oblivion” (Millière et al., 2018, p. 16). However, as Gallagher (2017, p. 5) argues, it is not at all clear how one can even report on these extreme states of consciousness without having registered them as one’s own (and not as someone’s else). To this argument, I further add that it does not matter whether the “one” these states refer to or are for, is myself, the world, the universe, everything or nothing, or whether this “one” implies a perspective centered onto a single point of origin inside myself rather than a perspective from everywhere or nowhere, or whether this “one” is embodied or fully disembodied. Actually, to be able to say that “I was the universe, I was everywhere and nowhere” or “(I forgot) that I was a male, a human, a being on Earth—all gone, just infinite sensations and visions” (reported by Millière et al., 2018), one must have been aware, while experiencing those extreme experiences, that they were experienced by oneself, whatever “oneself” or “I” refers to at the time of the experiences. Therefore, in my view, it is legitimate and safer to conclude that consciousness always implies at least a minimal level or form of self, even if some of its features can be missing.

With the term “affected” I refer not so much to the (more or less) permanent modifications that take place *after* the agent has experienced something and that are usually identified with “memories” and what was “learnt.” Nor do I generally refer to whatever (physical, chemical, etc.) changes may occur inside the agent’s organism. Rather, I specifically refer to the temporary effects that an agent’s given operation has on the agent’s *self*, that is, at the level that – by summarizing the complexity inherent to the composite structure of the agent’s organism – represents and stands for the agent in its entirety as a single unit. Most frequently, these effects imply a (temporary) variation in the state of the self, but sometimes they may imply no variation. This is reflected in our languages by verbs and nouns that allow us to express the conscious experience of a lack of change, and say for example that “we noted no differences,” or that “nothing happened” (for the sake of simplicity, we can use the term “variation” to generally refer to the effects that an agent’s operation has on the agent’s self, irrespectively of whether they imply a variation or not).

As an example of the possible effects that the agent’s operations have on its *self*, consider the experience of pain. This experience, metaphorically speaking, “tells” the agent that it is undergoing a specific variation that affects it as a whole, as a single unit, and that this variation is characterized by a certain intensity and a certain hedonic aspect that distinguish it from other types of variations. For example, the variation that the agent undergoes when it feels pain has an opposite hedonic aspect compared to pleasure: while the former acts as a “block” that forces the agent to operate in a different way (so as to remove the cause of the pain), the latter “sustains” the agent’s activities, leading it to keep on doing what it’s doing.

In their essence, these temporary variations represent the impact that the agent’s own (voluntary or involuntary) operations (such as perceiving, moving, thinking, remembering, dreaming, speaking, etc.) have on the agent’s self. They provide the agent with the direct, immediate and intuitive knowledge (on which rational knowledge can subsequently be built and developed) of how entities and events in general *relate* to the agent’s self: for example, how a certain object limits or facilitates the agent’s activity, how the agent can modify or use it, where the object is spatially located relative to the agent, etc. It is precisely these temporary variations that the agent’s self undergoes because of the entities and events with which the agent enters into relation, that allow the agent to define, represent, identify and recognize them.

In relation to this aspect, it is important to highlight that these temporary variations allow the agent to progressively build its personal knowledge not only about the entities and events it comes upon, but also about itself. Actually, as it has been observed (Rochat, 2003; Cleeremans, 2008; Ciaunica et al., 2021), the sense of self is not just given, but must be learnt and achieved: it emerges from the continuous process of differentiation between the agent and the other entities. It seems very plausible that, at least for humans, this differentiation process already starts *in utero*. The evidence reviewed by Ciaunica et al. (2021) shows that prenatal organisms possess a basic form of self-awareness. For example, fetuses spend a considerable amount of time in tactile exploration of the boundary between innervated and non-innervated areas. According to Ciaunica et al. (2021, p. 7), this demonstrates that “The fetus is thus exploring the boundaries of his or her self, developing knowledge of the effects of his or her own self-generated action, and its consequences.”

It must be further noted that the sense of self is not always explicitly experienced by the agent. Actually, most of the time when we experience something, we are not self-aware of it: we simply experience it without having the additional, explicit experience that it is we who are experiencing it. This does not mean however that on these occasions the sense of self is absent: in fact, it is present, but in a “pre-reflective” form. As it has been argued (Legrand, 2006, 2007; Gallagher and Zahavi, 2008), it is possible to distinguish between two forms of self-awareness: pre-reflective self-awareness and reflective self-awareness. The former is intrinsic, tacit, non-observational (i.e., not implying an introspective observation of oneself) and non-objectifying (i.e., it does not turn one’s experience into an observed object). The latter is explicit, observational and objectifying: it introduces a form of self-division or self-distancing between the reflecting and the reflected-on experience. Pre-reflective self-awareness is the constitutive structural feature of any conscious state: as such, it exists independently of reflective self-awareness; on the contrary, reflective self-awareness always presupposes pre-reflective self-awareness. Evidence shows that every conscious mental state always involves pre-reflective self-awareness: (i) as remarked by Husserl (1989, p. 18a), each thing that appears has *eo ipso* an orienting relation to us, even if we are just imagining it (if we are imagining a centaur, we cannot help but imagine it as in a certain orientation and in a particular relation to our sense organs); (ii) it is always possible for us to return to an experience we had and remember it as our experience, even if originally we did not live it explicitly as “our” experience. This would not be possible if the experience were completely anonymous, that is, lacking the property of intrinsically belonging to us; (iii) all our conscious experiences are given immediately as ours: we do not first have a conscious experience and only later the feeling or inference that it was ours!

Finally, it should be observed that the explanation I have put forward of the need of the PAC (“the PAC provides the agent with a sense of self, and informs it on how the self is affected by its own operations”), subsumes and can easily explain many of the answers that researchers and scholars have provided about the functions of consciousness, even if these answers were not originally intended to account for the functions of consciousness in terms of the PAC (see, for example, Baars, 1988; Morsella, 2005; Frith, 2010; Campbell, 2011; Earl, 2014; Keller, 2014; Pierson and Trout, 2017; Kanai et al., 2019). Let’s consider some of the most representative answers.

A very plausible answer by Kanai et al. (2019) is that experience has the function of internally generating “counterfactual representations” of events, that is, representations detached from the current sensory input, which enable one to detach oneself from the environment, simulate novel and non-reflexive behavior, plan future actions, and learn from fictional scenarios that were never experienced before. Similarly, for Earl (2014, pp. 13–14), organisms that possess only automatic responses may sometimes have no response to match a situation that confronts them, which could result in a missed opportunity or a risk to the organism; therefore, a mechanism, of which consciousness is a key component, has evolved to generate responses to novel situations. However, neither Kanai et al. (2019) nor Earl explain why only representations provided with the particular phenomenal aspect that consciousness assigns to them, allow us to simulate new behaviors and scenarios, plan future actions, etc. They only tautologically state that experiencing counterfactual representation allows you to experience new behaviors and scenarios, future plans, etc. The explanation I have provided, on the contrary, accounts for this by showing that one can simulate new behaviors and scenarios, etc., only if one can see the effects that these simulations have on oneself as a single unit, as a “single voice,” which primarily happens via the temporary changes one undergoes as a whole while mentally performing the simulations.

Another recurrent and plausible answer is that experience is adaptive (James, 1890/1983; Morsella, 2005; Earl, 2014). It is not a case that we developed unpleasant feelings toward what harms us and pleasant feelings toward what is good for us. If experience had no function at all, we could quite easily have developed unpleasant feelings toward what is good for us and pleasant feelings toward what harms us. More in general, if consciousness had no effects on behavior, it would not matter if our experiences were completely fantastical and had no correlation with reality (Earl, 2014, p. 7). However, scholars do not explain why just experience has this adaptive capacity, and leave the explanation to the reader’s intuition. My explanation, on the contrary, provides an answer to this question. Unpleasant feelings bring their action to bear on our behavior by inducing a temporary change in us that blocks us, in our wholeness, from doing what we are doing, and forces us to operate differently. In a similar but opposite way, pleasant feelings bring their action to bear on our behavior by inducing a temporary change in us that makes us continue to do what we were doing.

Morsella (2005) also provides another possible answer when he notes that the skeletal muscles - though often functioning unconsciously - are the only effectors that can be controlled directly via conscious processes. He argues that phenomenal awareness is needed to resolve conflicting, parallel impulses and cognitive processes in order to produce coordinated single actions by means of the skeletomotor system. In this view, consciousness acts as a forum that allows for information from different sources to interact in order to produce adaptive actions. Without consciousness, “the outputs of the different systems would be encapsulated and incapable of collectively influencing action” (Morsella, 2005, p. 1012). But why does just consciousness have this capacity to act as a forum? Morsella does not explain this. It is clear that Morsella’s argument rests on the presupposition that whatever impulse for whatever reason enters the forum of consciousness, is able to affect the agent in its entirety, not just a part of it. This can be realized only if there is a processing level that stands for the agent in its entirety and that allows the agent to understand the effect that the impulse has on it as a whole, which is precisely what my explanation suggests.

## The mechanisms that underpin the phenomenal aspect of consciousness

What is the mechanism that supports conscious information processing? According to my analysis (Marchetti, 2018), conscious information processing is made possible by two fundamental components: attention and what I have defined “the self” (from now on: S). Furthermore, a special role in the formation of complex forms of conscious experience is played by a sub-component of S: working memory (WM). These components are individually necessary and jointly sufficient for an agent to be conscious: taken individually, S and attention are fundamental parts of a conscious agent, but are not the same as a conscious agent considered in its entirety.

### The self (S)

S originates from the agent’s organism and comprises the agent’s body and brain (excluding attention and its organ): it is primarily expressed via the central and peripheral nervous systems, which map the agent’s body, environment, and interactions with the environment (Marchetti, 2018). It embodies all the competencies and abilities – physical, social, linguistic, and so on – the agent innately possesses and acquires in its life (at the end of which, S ceases to exist).

Besides providing the physical and material basis for all the agent’s organs, S supplies the *contents* of phenomenal experience: perceptible ones, such as “yellow” and “cat,” as well as intangible ones, such as memories, ideas, and emotions.

S runs the organism according to a fundamental principle or goal, which governs all the other principles: to stay alive. Operationally, the principle can be expressed as follows: “operate in order to continue to operate” (Marchetti, 2010). This is the vital instinct, the algorithm of life, which is already present in the simplest cell (Damasio, 2010).

This principle is primarily instantiated in a hierarchy of values, among which the biological ones (e.g., homeostasis) play a pivotal and foundational role. On these values other kinds of values (e.g., cultural) can be developed during the agent’s life. These values define what is relevant and meaningful for the agent, and guide the development of S.

The development of S occurs as a consequence of the agent’s activity, namely its interaction with the (natural and social) environment. The agent’s activity and its outcomes are mapped by the brain, which leads to a continuous modification of S. This process is differently described and termed by scholars: see for example Baars’s (1988) creation of new unconscious contexts, Edelman’s (1989) reentrant mechanisms, which allows for categorization and learning, and Damasio’s (1999) formation of first- and second-order brain maps.

S helps maintain and expand the well-being of the agent in its entirety: it provides a sufficiently stable platform and source of continuity relative to the outside world. As highlighted by Damasio (2010, p. 200), the working of its more or less stable parts (internal milieu, viscera, musculoskeletal system, etc.) constitutes an “island of stability within a sea of motion. It preserves a relative coherence of functional state within a surround of dynamic processes whose variations are quite pronounced.”

This “island of stability” is made possible mainly by the values on which S is centered: it represents the central, (almost) unchanging core of S that assures the continuity of the organism (and ultimately of the agent) across the various modifications that it can undergo. Moreover, this “island of stability” acts as a reference point that allows for the detection (by attention) of the relevant changes of the state of S that are occasioned by the agent’s activity and by the inner processes of the organism. The detection of these changes allows the agent to promptly react, according to the relevance they have for it. By means of the agent’s activity, the homeostatic range associated with well-being can thus be reestablished.

As I said before, S supplies the contents of phenomenal experience. But does consciousness actually require any content in order to occur? It could be argued that content is not a necessary condition for consciousness. With regard to this issue, various scholars (Thompson, 2015; Millière et al., 2018; Josipovic and Miskovic, 2020; Srinivasan, 2020) have reported cases of conscious experience of reduced or even absent phenomenal content. These cases can occur in several situations: when transitioning to and from sleep, when waking from anesthesia, under the influence of psychedelics, and during meditation. These cases seem to call into question the necessity of content for consciousness (but not of S, because S provides all the necessary material support for consciousness). However, upon a closer look, this conclusion turns out to be a bit premature. Let’s consider for example the contentless experience that marks the first instant of awakening: it is true that the only thing one feels is to be alive in the present moment (sometimes one does not even know who one is or where one is), but it is equally true that upon having it, one is automatically and unavoidably led to the more common kind of experience-with-content (“I am in my bedroom”) that characterizes daily life. This seems to indicate that experience-with-content is the unavoidable and unescapable default conscious state, and that experience-without-content is just a temporary, intermediate form of consciousness.

A final remark about the adequacy of my definition of S. As it is known, there is not much consensus on a common definition of the self. Various scholars and philosophical schools adopt different definitions of the self (Di Francesco and Marraffa, 2013; Facco et al., 2019). If we consider just the Western tradition, we can see a range of definitions that goes from those that deny the existence of the self – such as Hume (1739/1985), who claimed that the self is just a fictional entity, or Dennett (1991), for whom the self is an illusory construct – to those that admit its existence – such as James, who, described the spiritual self as something with which we have direct sensible acquaintance and is fully present at any moment of consciousness (James, 1890/1983, p. 286), or Strawson (1997, p. 424), who, without involving any conceptions of agency, personality and long-term diachronic continuity, defines the self as a single, mental thing that is distinct from all other things and is a subject of experience. In this context, I have devised my definition of S by basing it, as much as possible, on current scientific knowledge and empirically ascertained facts, and by following the principle of its functional usefulness in explaining the PAC. As such, S can be considered as an appropriate and comprehensive scientific construct. Obviously, as all constructs, it can be modified, improved or even abandoned in favor of other constructs if the latter prove to work better.

### Attention

S can be considered as the main step of the evolutionary process that reduces the complexity inherent to the composite structure of an organism into the “single voice” (Damasio, 2010) of a single entity – a reduction, which, as we have seen, helps the agent to behave in a coordinated manner and avoid conflicting responses. This process was mainly achieved through the activity performed by neurons and the nervous system, which allows for the creation of representational patterns (e.g., topographic maps, transient neural patterns) that are capable of mapping the agent’s activity.

The ultimate step of this process of reduction was phylogenetically achieved by attention and its direct product: conscious experience.

Attention is a mechanism^6^ (Kahneman, 1973, p. 2) that allows for the realization of a single “perspectival point” from which the agent can experience objects: whatever we perceive, think, etc. is always perceived, thought etc. from a unique perspective, and arrayed around this perspectival. This point makes attentional focusing always directed “toward something” and partitions the world into an asymmetric space that makes us perceive objects from our perspective. This is possible because attention is deployed from a single point inside our body, which, according to Merker (2013a, p. 9), “is located at the proximal-most end of any line of sight or equivalent line of attentional focus.”

The reduction process is further strengthened by the periodic nature of attention, which makes it possible to restrict conscious processing to temporally limited and distinct processing epochs (Pöppel, 1997, 2004: Wittmann, 2011). By framing one’s conscious experience on a temporal basis, one can reduce and divide the uninterrupted, chaotic and manifold stream of stimuli into basic units, real “building blocks” that can be used (with the support of WM and the other kinds of memory) to form ordered and more complex sequences (Marchetti, 2014).

The periodic (or “pulsing”) nature of attention has been empirically verified by a number of experiments that used behavioral, psychophysical or electrophysiological methods. The experiments showed that attention operates rhythmically at a frequency that ranges from 0.5 to 10 Hz approx. (VanRullen et al., 2007; Bush and VanRullen, 2010; Landau and Fries, 2012; Fiebelkorn et al., 2013; VanRullen, 2013, 2018; Song et al., 2014; Zoefel and Sokoliuk, 2014; Dugué et al., 2015; Landau et al., 2015; Fiebelkorn and Kastner, 2019; Senoussi et al., 2019; Zalta et al., 2020).

Finally, attention further enhances the reduction process by allowing the agent to select just one or a very few elements, and suppress the other stimuli. The selection process can variously occur: attention can be deployed exogenously or endogenously (Theeuwes, 1991, 2010; Connor et al., 2004; Carrasco, 2011; Chica et al., 2013; Katsuki and Constantinidis, 2014), internally or externally (Chun et al., 2011), spatially (Posner, 1980; Posner and Cohen, 1984), at variable levels of intensity (La Berge, 1983) and for variable amounts of time (La Berge, 1995), at variable levels of size (narrowly or widely) (Treisman, 2006; Demeyere and Humphreys, 2007; Alvarez, 2011; Chong and Evans, 2011), simultaneously between central processes and peripheral processes, as well as between different perceptual modalities (Pashler, 1998).

This has led Tamber-Rosenau and Marois (2016) to conceptualize attention as a structured mechanism arranged in various levels and parts having different functional roles, such as: a central level for abstract, cognitive processes, a mid-level containing priority maps that bias competitions in representational formats and sensory modalities, and a peripheral level for sensory processes.

### Working memory

The basic “building blocks” shaped by attention can be combined and assembled by WM, in order to form longer and more complex experiential sequences.

Working memory maintains information in a heightened state of activity in the absence of the corresponding input over a short period, in order to allow for its manipulation during ongoing cognitive processing. This makes it possible for the agent to perform various kinds of operations, from relatively simple ones – such as comparing two items, constructing an item using another item as a model – to more complex ones, such as flexibly combining elements into new structures (Oberauer, 2009), imagining future events (Hill and Emery, 2013) and integrating information from the past into representations of the present or future (Hasson et al., 2015; Parr and Friston, 2017).

Working memory also helps to correctly discriminate relevant from irrelevant information, by preventing the interference of automatic tendencies and routines (Unsworth and Engle, 2007).

Neuroscientific studies have started to elucidate the possible mechanisms underlying WM (Fingelkurts et al., 2010; Lisman and Jensen, 2013; Roux and Uhlhaas, 2014). For example, according to Roux and Uhlhaas (2014), it is the cross-frequency coupling (CFC) between theta, alpha and gamma oscillations that underpins WM activity. Gamma-band oscillations would reflect a generic mechanism for active maintenance of WM information, theta-band oscillations would be involved in the temporal organization of WM items, and oscillatory activity at alpha frequencies would play a critical role in protecting WM items from non-relevant information. CFC between theta- and gamma-band oscillations would “provide a code for representing multiple and sequentially ordered WM items in which cycles of gamma-band oscillations are coordinated through an underlying theta rhythm” (Roux and Uhlhaas, 2014, p. 22). On the contrary, CFC between gamma and alpha oscillations would be involved in the maintenance of sensory-spatial WM items.

### Conscious information processing is produced by the interaction between attention and S

**FIGURE 1:**
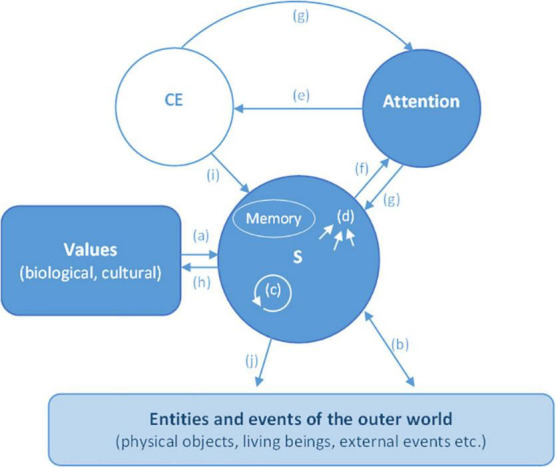
Conscious information processing: its main component parts. *S (the self*): S develops and works on the agent’s innate biological and culturally acquired values **(a)**. The interactions between S and the outer world **(b)**, the inner processes of S (e.g., routines automatically triggered by unconscious perception or by conscious experiences) **(c)** and the memory system (long term memory, working memory, procedural memory, etc.) usually induce changes in the state of S **(d)**, which provide the content for attentional processing (but the content can also be represented by the absence of any change). *Attention*: Attentional processing produces **(e)** conscious experience (CE). Attention can be stimulus, bottom–up driven **(f)** or can be voluntarily, top–down directed according to the agent’s consciously processed goals **(g)**. *Conscious experience (CE*): Conscious experience engenders temporary or permanent modifications of S (via the memory system) **(i)**, pilots attention **(g)**, triggers intentional actions **(j)**, unconscious processing **(c)**, and induces modifications of cultural values **(h)**.

Conscious information processing is produced by the interaction between attention and S, when the state of S is focused on by attention. Before such an interaction, there is no consciousness: consciousness only emerges from it^7^. The state of S provides the content of attentional processing and consequently of consciousness. Usually, attention focuses on and enhances the changes of the state of S, and mainly those that are physically salient, or most relevant for the agent’s current goals or selection history (what the agent has learnt in the past: Awh et al., 2012), or for the maintenance of the agent’s homeostatic values. However, the content of attentional processing can also be represented by the absence of any change of the state of S (see Figure 1).

The changes of the state of S can be generated endogenously, such as when the level of our blood sugar drops or exogenously, such as when an object attracts our attention. They can be directly induced by a voluntary decision, such as when we purposefully think about something, or indirectly triggered as part of a routine action. The kind of change depends on the structures and levels of S that are involved by the change. For example, when we interact with physical objects, changes can occur at the levels of the specialized sensory system involved (touch, smell, etc.), but also of the musculoskeletal system. The changes of the state of S can have various durations, from short intervals of the orders of milliseconds to long intervals of the order of several seconds. Sometimes, these changes can induce automatic reactions intended to reestablish the homeostatic range, but they can also require no specific corrective activity by the agent.

It is important to note that not always what is focused on by attention becomes conscious: actually, there can be attention without conscious experience (Naccache et al., 2002; Montaser-Kousari and Rajimehr, 2004; Sumner et al., 2006; Bahrami et al., 2008).

Some scholars (Lamme, 2003; Koch and Tsuchiya, 2006; van Boxtel et al., 2010; Bachman, 2011) have gone so far as to claim that there can also be consciousness without attention. However, as highlighted by various scholars (Srinivasan, 2008; Kouider et al., 2010; Marchetti, 2012; Pitts et al., 2018; Munévar, 2020; Noah and Mangun, 2020), this claim seems to result from a wrong interpretation of the experimental data, which originated from not having considered the various forms and levels that attention (Nakayama and Mackeben, 1989; La Berge, 1995; Lavie, 1995; Pashler, 1998; Treisman, 2006; Demeyere and Humphreys, 2007; Koivisto et al., 2009; Alvarez, 2011; Chun et al., 2011; Tamber-Rosenau and Marois, 2016; Simione et al., 2019) and consciousness (Tulving, 1985; Edelman, 1989; Iwasaki, 1993; Bartolomeo, 2008; Vandekerckhove and Panksepp, 2009; Northoff, 2013; Northoff and Lamme, 2020) can assume. In fact, not all forms of attention produce the same kind of consciousness, and not all forms of consciousness are produced by the same kind of attention; there can be kinds of conscious experience with no top–down attention but with bottom-up attention; there can be kinds of conscious experience in the absence of a focal form of top–down attention but in the presence of a diffused form of top–down attention. In sum, there can be cases of attention without consciousness, but never cases of consciousness in complete absence of some form of attention: attention is necessary for consciousness.

Complex forms of conscious experiences, such as the various modes of givenness of conscious experience and the stream of consciousness, require the support of the memory system, and notably, of WM. WM allows for the combining and assembling of the basic pieces of information that are isolated and shaped by attention.

Incidentally, it should be noted that for some researchers, the activity of WM can be ultimately traced back to the working of attention: WM functions would emerge when attention, being internally oriented toward the neural systems that were originally involved in the processing of the object/event to be remembered, allows for their recruitment and activation, and consequently for the re-processing of the object/event (Postle, 2006; Lückmann et al., 2014).

What the agent consciously experiences can have various kinds of consequences for the agent: for example, it can lead the agent to voluntarily perform some actions, modify its acquired cultural or social values, or perform further unconscious processes. Importantly, conscious experience usually triggers adaptation and learning processes that lead, via the memory system, to more or less permanent changes of S. Once implemented, these changes alter the way the agent’s brain processes information: for example, repeated processing of a stimulus leads to habituation, and repeated practice to automatization of the practiced skill (Baars, 1988). This implies that an agent never experiences the same object twice in the same way because the relationship between it and the object undergoes continuous transformations. One of the most relevant consequences of such changes is the development of reflective self-awareness, which fundamentally enhances the agent’s autonomy by allowing it to set its own objectives and directly control its own behavior. Incidentally, it should be noted that there are cases in which conscious processing does not trigger any learning process, such as in the case of amnesic patients (Damasio, 1999), who, despite exhibiting conscious behavior, are unable to learn any new fact.

### Phenomenal aspect of consciousness production: Attentional activity and the modulation of the energy level of the organ of attention

What is the process that allows attention to render the state of S conscious, that is, to assign it the phenomenal aspect characteristic of conscious experience (the PAC)? According to my hypothesis (Marchetti, 2010, 2018), (voluntary or involuntary) attentional activity (AA), by focusing on and enhancing the (changes or absence of changes of the) state of S, engenders a modulation of the energy level of the neural substrate that underpins AA itself: *it is precisely this modulation that produces the PAC*.

My hypothesis is based on the assumptions that:

(a) What makes AA possible is the neural energy provided by the neural substrate that constitutes the organ of attention (OA);
(b) The detection of the state of S by means of AA modulates the energy level of the OA.

More specifically, given that attention can be considered a structured mechanism that is arranged in various levels and parts having different functional roles (Tamber-Rosenau and Marois, 2016), the OA can also be considered as structured in various levels and parts, each supporting these different roles. Consequently, the modulation affects only those levels and parts of the OA (from now on, “OA area”) that underpin the detection of the state of S.

My assumptions are based on a number of observations and evidence.

The idea that attention is based on an energy pool has a consolidated history. It was first put forward by Kahneman (1973), on the footsteps of David Rapaport. Although initial research seemed to show the existence of a “general-purpose” energy pool, subsequent experiments have shown that there are a variety of resources that are “task specific” (McLeod, 1977; Duncan, 1984; Pashler, 1989). Various psychological experiments and observations clearly show that such a pool is limited: the possibility of sharing attention is limited by the task demands: when one task demands more resources, there will be less capacity left over for the other tasks (Lavie, 1995); there is a limit to increasing mental processing capacity by increasing mental effort and arousal; an extensive use of attention, as demanded by complex, time-consuming tasks, requires some time to recover the consumed energy; etc.

The concept of an “organ of attention” is not new: many scientists have already started investigating the neural and brain structures constituting it (Mesulam, 1990; Posner and Petersen, 1990; Crick, 1994; Crick and Koch, 2003). However, the search for such an organ is not fully uncontroversial. As De Brigard (2012) highlights, there is disagreement as to the nature of the neural correlate of attention: some scholars suggest that there may not be a single neural process responsible for all forms of attention (Wu, 2011), while some others see attention as a unified cognitive process with an identifiable sub-personal neural correlate (Prinz, 2011). Undoubtedly, only a clear definition of the features and roles of attention can help define the nature of its organ.

The concept of neural energy has been prevalently studied with regard to its consumption (in terms of demand of adenosine triphosphate, ATP) during neural informational processes, that is, for its support function in information processing (Laughlin et al., 1998; Laughlin, 2001; Laughlin and Attwell, 2004; Shulman et al., 2009; Sengupta et al., 2010). Recent studies have started investigating how to decode the information of stimulus and neural response from the energy metabolism (Wang et al., 2017). However, to my knowledge, no empirical work has been conducted so far to investigate neural energy in connection with AA as I have theorized it.

The concept of energy has been explicitly associated with consciousness in recent studies (Street, 2016; Pepperell, 2018). However, these studies tackle preferentially the how of the PAC – how it is brought about – rather than the why of the PAC: Street highlights that consciousness and its major features derive from an efficient use of energy and the maximization of thermodynamic efficiency (“self-awareness may be a mechanism for optimizing the brain’s consumption of energy”) and Pepperel focuses on how conscious experience is brought about by a certain organization of the energetic activity in the brain (conscious experience is caused by “a certain dynamic organization of energetic processes having a high degree of differentiation and integration”).

The idea that AA engenders a modulation of the energy level of the OA primarily derives from the observation of the extreme consequences that such a modulation can bring about, such as when the normal flow of attention is dramatically slowed down or even interrupted. This is the case of pain. A nociceptive signal captures attention. This engenders a modulation of the energy level of the OA that, in the case of acute or persistent pain, can lead to an interruption of the normal flow of attention (so much so that, in order to reestablish the normal state, we must either divert our attention toward something else or try to remove the cause of the pain) (Eccleston and Crombez, 1999; Haikonen, 2003; Legrain et al., 2009) – which is precisely what the experience of pain consists in.

It is important to highlight that the working of the OA, like the working of any other organ of the organism, depends on the energy supplied by the organism. To work properly, the OA needs a certain amount of energy. The amount of energy needed by the OA can vary according to various factors, such as the agent’s expectations and motivations, and the task that the agent has to perform. It is my hypothesis that the amount of energy that the organism supplies to the OA determines the agent’s state of arousal (or wakefulness). Various states of arousal are possible (some of which can also be induced pharmacologically): conscious wakefulness, REM sleep, deep sleep, vegetative state, near-death experience (NDE), coma, etc. (Laureys, 2005; Laureys et al., 2009). One of these states – NDE – is particularly interesting, because it apparently represents a challenge to physicalists theories of mind and consciousness. Greyson (2000, pp. 315–316) defines NDEs as “profound psychological events with transcendental and mystical elements, typically occurring to individuals close to death or in situations of intense physical or emotional danger.” Prototypical features of NDE are out-of-body experiences (OBE), experiencing a panoramic life review, feeling of peace and quiet, seeing a dark tunnel, experiencing a bright light (Vanhaudenhuyse et al., 2009; Martial et al., 2020). While some scholars believe that it is possible to explain NDEs in psychological or neurobiological terms (see for example Mobbs and Watt, 2011; Martial et al., 2020), some other scholars argue that physicalists theories of the mind cannot explain how people can experience the vivid and complex thoughts of the NDE, given that brain activity is seemingly absent (see for example Haesler and Beauregard, 2013; van Lommel, 2013). I think that the theoretical framework proposed by Martial et al. (2020), which is compatible with my model of consciousness, and their analysis of NDE, can help to define how the brain generates NDE without postulating any paranormal cause. According to Martial et al. (2020), consciousness has three main components – wakefulness, connectedness (akin to external awareness) and internal awareness –, which allow for mapping the various states of consciousness. In a normal conscious awake state, the three components are at their maximum level, while states such as coma and general anesthesia have these three components at their minimum level. NDE corresponds to internal awareness with a disconnection from the environment experienced in unresponsive conditions. In terms of my model, this means that attention is deployed only internally and that the amount of energy that the organism supplies to the OA is almost negligeable, albeit sufficient for OA to support some (minimal) kind of AA.

## The main dimensions of the phenomenal aspect of consciousness and their relation to the modulation of the energy level of the organ of attention area

As I said, according to my hypothesis, the PAC is brought about by the modulation of the energy level of the OA area that is consequent upon the (voluntary or involuntary) use of attention. The PAC can be qualified according to at least five main dimensions: qualitative, quantitative, hedonic, temporal and spatial (see also Cabanac, 2002, who however does not include the spatial dimension). Each dimension can be traced back to a specific feature of the modulation of the energy level of the OA area (see Table 1).

**TABLE 1:** PAC dimensions, how they relate to the modulation of the energy level of the OA area, and the features of the sense of self involved.

PAC dimension	Features of the modulation of the energy level of the OA area that define the PAC dimension	Features of the sense of self involved
Qualitative	OA area involved by the modulation	Single voice
Quantitative	Amount of variation of the energy level	Single voice
Hedonic	Direction of variation of the energy level relative to the set-point at which the level of the OA area is regulated	Boundaries of the self and sense of mineness
Temporal	Periodicity of the modulation of the energy level	Feelings of continuity; single voice
Spatial	Path followed by the modulation of the OA	Point of view; single voice

The qualitative dimension of the PAC is defined by the OA area that, underpinning the attentional processing of the state of S, is modulated by such an attentional processing. This means that what an agent consciously experiences about the state of S also depends on the way the agent attentionally processes the state of S (and consequently on the areas of the OA involved), rather than on the state of S alone. In fact, the same state of S may undergo different levels of attentional processing, which lead to different conscious experiences of the state itself (affective, cognitive, sensory, etc.)^8^.

The quantitative dimension is defined by the amount of variation of the energy level of the OA area caused by the modulation.

The hedonic dimension (e.g., pleasant vs. unpleasant) is defined by the direction of the variation of the energy level of the OA area relative to the set-point at which the level of the area is regulated^9^. Pleasant and unpleasant experiences occur when the energy level moves toward or away from the set-point, respectively. More precisely, painful experiences take place when the energy level moves away from the set-point beyond a certain threshold. When this occurs, the agent’s flow of attention is diverted from any ongoing task and is fully absorbed by the painful stimulus and its possible causes, so that the agent can take the necessary actions to restore the original energy level of the OA area. Pleasant experiences occur when the energy level of the OA area is restored to its original value after it was brought beyond a certain threshold. Neutral experiences – or “comfort” as defined by Cabanac (2013), a state characterized by physiological normality and indifference toward the environment -, occur when the energy level fluctuates within an acceptable range of the set-point.

Incidentally, it is interesting to note that Solms (2019) has proposed a similar mechanism for affect (the technical term for feeling). Solms identifies affect as the elemental form of consciousness, which has its physiological mechanism (an extended form of homeostasis) in the upper brainstem. Affect enables complex organisms to register, regulate and prioritize deviations from homeostatic settling points in unpredicted contexts. Deviations away from a homeostatic settling point is felt as unpleasure, and returning toward it is felt as pleasure. Solms’ proposal very much resembles my proposal in that it explains the hedonic dimension in terms of deviations to and from a set-point (but this is not the only point of resemblance: it also stresses the importance of investigating the function of conscious experience to overcome the explanatory gap, and poses a fundamental biological imperative – to minimize expected free energy – at the basis of the existence and survival of self-organizing systems). However, his proposal substantially differs from mine because it explains affects in purely homeostatic terms rather than in attentional ones (as deviations to and from the set-point at which the level of the OA area is regulated). In my view, Solms’ proposal precludes the possibility of explaining how the various kinds of variations of the self (chemical, electrical, mechanical, etc.) can be translated into the “common language” of consciousness: a translation that is made by attention and that makes it possible to compare and differentiate the various dimensions of life. Most probably, this limit of Solms’ proposal derives from his overestimation of the role of brainstem as the primary mechanism of consciousness, and underestimation of the role played by other mechanisms (this has also been observed by Safron, 2021).

The information provided by how the energy level of the OA area varies relative to the set-point at which the level of the area is regulated is fundamental for building the sense of mineness (or ownership) and defining the boundary between self and non-self. Considering for example the set-points related to homeostatic regulation, a departure of the energy level from the set-point indicates a departure from what is under the control of the agent. Some other mechanisms were proposed to account for the sense of mineness and the distinction between self and world, such as the comparator model (Gallagher, 2000; Legrand, 2006). However, as pointed out by Vosgerau and Newen (2007), these models presuppose the self-world distinction rather than explaining it. Actually, the agent, in order to learn the effects of its own movement, must already know which of its movements is caused by itself and which is not (for further criticisms of the comparator model, see Synofzik et al., 2008): a knowledge that, in my view, can only be provided by the hedonic dimension.

The temporal and spatial dimensions of the PAC are determined by the manner in which attention works. The temporal dimension of the PAC is determined by the periodic nature of attention. As we have seen, attention works in a periodic manner. On the one hand, this limits the duration of the modulation of the energy level of the OA and consequently of any conscious experience. On the other hand, it represents the necessary condition for the activity of modulation to be repeatedly performed, and consequently to produce – with the support of WM – the feeling that our experience flows uninterruptedly.

The spatial dimension of the PAC is determined by the egocentric spatial nature of attention. Every attentional pulse originates and is deployed from a single point located inside our body, and is directed toward something. Consequently, whatever is focused by attention, appears in our consciousness as possessing a spatial quality that is defined through the center of attention and the direction toward which attention is focused. The path that attention takes at every new cycle of its activity is reflected in the OA area that underpins and is modulated by the activity performed by attention. The modulation of the OA follows the path taken by attention: it starts from the point where attention originates and continues to the point where the deployment of attention stops.

A clarification is in order concerning the temporal and spatial dimensions. These features of the PAC must not be confused with the conscious experience of time and space, respectively. One thing is the experiences of time and space, quite another the temporal and spatial dimensions of experience. You can consciously experience something (e.g., an emotion) without experiencing or being aware of the temporal or spatial dimension of your experience. The temporal and spatial dimension of the PAC are a precondition for any experience to occur^10^, including the experiences of time and space, but they are not in themselves experiences of time and space. For such experiences to occur, a specific assembling – performed with the support of WM – of the contents selected by attention is necessary (Marchetti, 2014)^11^.

A final consideration concerning the evolutionary origins of consciousness: did all the five dimensions of the PAC appear together at the same time, or did one or some of them appear before the others? If we adopt the evolutionary transition marker adopted by Bronfman et al. (2016) (unlimited associative learning) or the neurobiological features of consciousness listed by Feinberg and Mallatt (2013) and Feinberg and Mallatt (2019) as criteria to define the appearance of consciousness, it seems quite reasonable to conclude that all the five dimensions of the PAC emerged phylogenetically together at the same time (obviously, because of the different sensory and brain machinery with which different species are endowed, the five dimensions can differ between the various species: for example, what a fly sees, is qualitatively different from what we humans see Lamme, 2018). However, stricter criteria can lead to different conclusions.

## Conclusion

In this article, I have put forward an explanation of the difference that the PAC makes for information processing and for the agent processing it. My view is that the PAC supplies the agent with a sense of self, and informs the agent on how its self is affected by its own operations. This has many advantages for the agent, among which the most relevant are that the agent can: see itself as an entity among, and differentiated from, other entities; build a knowledge of how other entities and events refer to itself; build a knowledge about itself and ultimately develop a form of reflective self-awareness; produce coordinated behaviors and avoid conflicting actions that could damage its integrity. In turn, this allows the agent to (at least up to a certain point) set its own goals and avoid automatic responses, act independently from the influence of its natural and social environment, build an autonomous knowledge by resisting possible wrong information, and on that basis, form justified, supported beliefs: in a word, to dramatically enhance the agent’s autonomy (Castelfranchi, 2012).

The PAC performs its two main functions (providing the agent with a sense of self, and informing the agent about how the agent’s self is affected by the agent’s own operations) through its five main dimensions: qualitative, quantitative, hedonic, temporal, and spatial.

As to the sense of self, we have seen that it provides the agent with the feeling of being an entity differentiated from other entities, the presence of a “point of view,” the capacity to represent itself with a “single voice” and the feeling of continuity. Each of these features is shaped through the five dimensions of the PAC (see Table 1). The hedonic dimension, by signaling how much the energy level of the OA deviates from the set-point, contributes to defining the boundaries of the agent and the sense of mineness; the spatial dimension provides the point of view; the qualitative and quantitative dimensions, associated with the limited temporal duration of any conscious experience and the point of view, make the “single voice” possible; the temporal dimension provides the feeling of continuity.

As for the information concerning how the agent’s self is affected by the agent’s own operations, we have seen that it is made possible by the modulation of the energy level of the OA area that is caused by AA. This modulation affects, both directly and temporarily, the agent’s self along some or all of the five dimensions of the PAC. Usually, the most affected dimensions are the qualitative, quantitative and hedonic ones, even though sometimes the spatial and temporal dimensions can be affected as well: for example, novel events seem to last longer the first time they are experienced than the subsequent times, while when witnessing unexpected, dangerous or shocking events, we are induced to perceive time as slowing down, etc.

Even though part of the hypothesis I have put forward in this article is based on empirical evidence, much remains to be experimentally verified: principally, the causal relation between the variations of the energy level of the OA area and the PAC. This preliminarily requires the exact identification and delimitation of the OA and of its various parts, and the possibility to measure its energy level. Moreover, even though it seems intuitive that, in the operative closure of an organism, AA may engender a variation in the energy level of the OA, the existence of such a direct relationship needs to be fully ascertained.

An empirical verification of the hypothesis can also be obtained by using it to build an artificial conscious machine. Among other things, this would allow for accepting or rejecting the opposite claim that a machine (e.g., a robot) that is equipped with S and attention and that displays the five dimensions of PAC, cannot have any conscious experience. To this end, in my view what counts most is that the concepts (and the relations among them) used to describe the hypothesis, can be operationalized, and that they allow one to analyze consciousness in terms of functions that are performed by the working of physical organs.

Finally, my hypothesis is partly compatible with those scientific approaches that conceive consciousness as the result of the nested and synchronized oscillatory neural activity across different time scales, such as Operational Architectonics (Fingelkurts et al., 2010) and Temporo-spatial Theory of Consciousness (Northoff and Huang, 2017). Even though these approaches do not directly address the why of the PAC and do not consider attention as the primary mechanism for consciousness, they account – as my proposal does – for the periodic and transitory nature of conscious processing, for the combinatorial capacity of the brain and for how conscious contents and forms are determined by the state of ongoing oscillatory neural activity.

## Data availability statement

The original contributions presented in this study are included in the article, further inquiries can be directed to the corresponding author.

## Author contributions

The author confirms being the sole contributor of this work and has approved it for publication.

## Footnotes

1 Information has been considered a fundamental dimension of living beings (Roederer, 2003; Nurse, 2008; Skyrms, 2010; Farnsworth et al., 2013; Walker and Davies, 2013; Adami, 2015; Baluška and Levin, 2016), if not actually the fundamental dimension that distinguishes living beings from other entities. For example, Skyrms (2010, p. 32) states that: “it is the flow of information that makes all life possible.”

2 I use “information processing” in a very general sense here, which includes all the various operations that can be performed on and with information, such as generation, encoding, decoding, management, storage, transduction and transmission of information.

3 For a similar view, see Safron (2020, p. 22): “If *cognition* is primarily discussed in the abstract, apart from its embodied–embedded character, then it is only natural that explanatory gaps between brain and mind should seem unbridgeable.”

4 For the first part of the answer (“the PAC supplies the agent with a sense of self”), I have drawn upon Damasio’s (1998, 1999, 2010) work. See for example Damasio (1998, p. 1880): “what we must explain if we are to address the issue of consciousness is the generation of a sense of self and the generation of the sense that such self is involved in the process of perceiving the stimulus.”

5 As Mangan (2001) shows, these kinds of conscious experiences serve precise purposes. For example, the feeling of familiarity signals that what we are experiencing now has been encountered before.

6 It should be noted that this is not the only definition of attention given by scholars. As Styles (1997) observed, attention is not a unitary concept and there is disagreement as to what its nature is: for example, Anderson (2011) discards the causal conception of attention in favor of an effect account of attention. I adopt the definition of attention as a mechanism because, among the various definitions that are empirically plausible, it is the most functional to the hypothesis I put forward in this article.

7 Philosophers distinguish between strong (or radical) emergence and weak emergence (Searle, 1992; Chalmers, 2006; Searle calls them emergence2 and emergence1, respectively). A strong emergence view claims that consciousness cannot be deduced from the domain from which it arises: that is, the causal interactions between elements of the brain cannot explain the existence of consciousness. Consequently, such a view leads to the idea of an explanatory gap between consciousness and the brain. On the contrary, a weak emergence view claims that consciousness can be explained as the product of brain processes (see Feinberg and Mallatt, 2020, for a convincing argumentation - from a biological and neurobiological perspective – of the plausibility of the weak emergence view). What I am proposing is a weak type of emergence.

8 See also Northoff and Lamme (2020, p. 579), who, even if they adopt a different model of consciousness, recognize that: the “very same contents may undergo different levels of processing and different functions like sensory, cognitive, affective, which modulates these contents allowing us to access them in different ways.”

9 Set-points, even if innately determined, can – up to a certain limit - be adjusted by the agent according to its plans, goals, motivation, etc. Consequently, a stimulus such as a non-painful thermal one can be perceived either as pleasant or unpleasant according to the internal thermal state of the agent (Cabanac, 1971, 2006).

10 See for example Koivisto et al.’s (2009) experiments, which clearly show that spatial attention is a prerequisite for any conscious experience to occur, and Donovan et al. (2017), who show that spatial attention is necessary for object-based attention.

11 According to my hypothesis (Marchetti, 2009, 2014), this same construction principle, which involves attention and WM, has allowed human beings to build – starting from the basic, psychological experiences of time and space – more abstract concepts of time and space, such as the time and space of physics. In this view, the time and space of physics are derivative on the psychological experiences of time and space. This is evidenced by the fact that everything we know is known primarily in and through our conscious experiences: only successively can we “abstract” or rationalize our experience, and develop those *entia rationis* that characterize physics as well as the other sciences. As Vicario (2005, p. 13) observes : “The vocabulary of physics derives from everyday language, which describes direct experience, that is, psychological experience.”
